# Effects of sugar-free polyol chewing gums on gingival inflammation: a systematic review

**DOI:** 10.1007/s00784-022-04729-x

**Published:** 2022-10-14

**Authors:** Eva Söderling, Kaisu Pienihäkkinen, Ulvi Kahraman Gursoy

**Affiliations:** 1 Institute of Dentistry, University of Turku, Lemminkäisenkatu 2, 20520 Turku, Finland; 2 Department of Periodontology, Institute of Dentistry, University of Turku, Lemminkäisenkatu 2, 20520 Turku, Finland

**Keywords:** Xylitol, Sorbitol, Maltitol, Chewing gum, Gingival inflammation

## Abstract

**Objectives:**

A systematic review of published data was conducted with the aim of assessing the effects of sugar-free polyol chewing gums on gingival inflammation.

**Materials and methods:**

Electronic and hand searches were performed to find clinical studies concerning the effects of sugar-free chewing gums on gingival scores. Prospective randomized controlled clinical trials published between 1971 and 2021 were included in the review.

**Results:**

The initial search identified 46 erythritol, 102 xylitol, 23 sorbitol, and nine maltitol chewing gum articles. After applying inclusion and exclusion criteria, seven xylitol chewing gum studies, one sorbitol, and one maltitol chewing gum study with either high or fair quality were reviewed. In five out of the seven xylitol studies, xylitol gum decreased gingival scores. In two studies, xylitol decreased gingival scores compared to a polyol gum, and in three studies compared to no gum/gum base. As for sorbitol and maltitol, only sorbitol gum chewing showed a small decrease in gingival scores compared to the controls.

**Conclusions:**

Habitual xylitol gum chewing may reduce gingival inflammation. The low number of studies and their heterogeneity provide clear indications that the effects of sugar-free polyol chewing gums on gingival inflammation need further, well-controlled studies.

**Clinical relevance:**

Sugar-free chewing gums, especially xylitol gum, may function as adjuncts to toothbrushing for reducing gingival inflammation, but the evidence so far is inconclusive.

## Introduction

Chronic and uncontrolled gingival inflammation in response to dysbiotic dental plaque accumulation is considered key factor in the onset of periodontitis [1]. Several intrinsic and extrinsic factors, such as salivary flow and composition, and frequent carbohydrate consumption, influence plaque accumulation [2]. These “disease drivers” are also crucial for the symbiotic and dysbiotic character of oral microbial biofilms. In recent years, research has focused on ways to increase resistance of the microbiota to dysbiosis [3]. However, it is also true that plaque accumulation by itself increases the risk of dental disease. During the shift from periodontal health to gingivitis, bacterial biomass increases several-fold, increasing the number of all biofilm bacteria. Yet the influence of bacteria on gingival inflammatory response is more prominent especially for enriched species in the biofilm [4]. Therefore, since mechanical control is not always sufficient to disrupt and eliminate pathogenic biofilms, any adjuncts to the mechanical control of biofilm accumulation should help in preventing gingival inflammation [5].

During the past decades, a wide range of anti-plaque, anti-biofilm, and anti-inflammatory agents have been proposed as part of oral hygiene measures. Among them, plant-based essential oils [6] and probiotic bacteria [7] are the most studied group of adjunctive agents. Also polyol chewing gums have been suggested to function as adjuncts to routine oral hygiene measures such as toothbrushing [8]. Despite the high number of clinical studies with promising outcomes, factors such as the heterogeneities of intervention protocols and clinical evaluation methods limit the implementation of these adjunct therapies to clinical guidelines world-wide.

When it comes to oral health, all polyols are often regarded as similar, inert substances [9]. There are, however, major differences between polyols with respect to, for example, how oral microbes are able to utilize them, how they affect the composition of the oral flora, and how they influence plaque accumulation. Polyols based on six-carbon units, such as sorbitol and maltitol, are slowly fermented by oral microorganisms, and *mutans streptococci* can even be adapted to ferment them [10]. The four-carbon polyol, erythritol, and the five-carbon polyol, xylitol, are not fermented by oral microorganisms [10, 11]. In addition, erythritol and xylitol inhibit growth and biofilm formation of *mutans streptococci* in vitro [12, 13]. Clinical studies with sugar-free polyol chewing gums are generally conducted with gums with xylitol, sorbitol, or maltitol as the main sweeteners. To our knowledge, there are no clinical studies with erythritol chewing gum. Several clinical studies indicate that the habitual consumption of xylitol chewing gum decreases the counts of *mutans streptococci* [14]. Most studies agree that sorbitol gum chewing does not affect *mutans streptococci* levels significantly [14]. Maltitol consumption has both decreased *mutans streptococci* counts [15] and showed no effects [16]. In clinical studies, xylitol and sorbitol gum consumption appeared to have little influence on the composition of the oral microbiota [17–19]. For maltitol gum, both changes [20] and no changes [21] in the plaque microbiota have been reported. In our recent review, we demonstrated that xylitol gum chewing is likely to decrease plaque formation significantly [22]. Also sorbitol and maltitol gum may reduce plaque accumulation; however, the effects are usually small [22, 23]. To our knowledge, there are no studies on the effects of polyol gum consumption on levels of periodontal pathogens.

Sugar-free gum is recommended by several organizations, for example, the American Dental Association (ada.org). While these recommendations usually focus on reducing the risk of caries occurrence by chewing gum, it is indeed true that sugar-free gum may also significantly benefit gingival health. To our knowledge, only one systematic review concerning the relation between sugar-free polyol gums and clinical indices of gingival inflammation has been published [23].

With this systematic review we wanted to answer the defined research questions: (1) can the consumption of sugar-free polyol chewing gums reduce gingival inflammation, and (2) are there any differences in the effects of various polyols/polyol mixtures? To achieve this, we described and evaluated the literature published during 1971–2021 concerning the effects of sugar-free polyol chewing gums on indices of gingival inflammation.

## Materials and methods

The Preferred Reporting Items for Systematic Reviews and Meta-Analyses (PRISMA 2020 Statement: prisma-statement.org) was used as a guideline in the present systematic review. The review was registered in PROSPERO (CRD42020219021) before the data collection started.

### Information sources and search strategies

The research question for the present systematic review was formulated using PICO characteristics (Patients, Intervention, Control, Outcome), as follows: in healthy subjects (P), is sugar-free polyol chewing gum (I), compared with a control (no gum, gum base, a polyol gum) (C), effective in decreasing gingival inflammation (as measured by clinical indices) (O)?

The systematic review to identify all the relevant studies published was conducted from three databases: PubMed, Embase, and the Cochrane Library. Gray literature was searched on ClinicalTrials.gov. A hand search was conducted in the reference list of previous systematic reviews close to the topic [23, 24]. The searches were conducted on December 27–29, 2021, and checked for additional literature on January 24–25, 2022.

The following terms were used in the search for xylitol studies:

(xylitol* OR “xylitol”[Mesh]) AND (gingivitis* OR “periodontitis”[Mesh] OR “bleeding on probing*” OR “gingival bleeding*” OR “pocket depth*” OR “gingival index*” OR “gingival score” OR “Periodontal Index”[Mesh] OR “periodontal index*” OR (gingival* AND “index score*”))—PubMed.

(xylitol* OR “xylitol”/exp) AND (gingivitis* OR “bleeding on probing*” OR “gingival bleeding*” OR “pocket depth*” OR “gingival index*” OR “periodontal index*” OR (gingival* AND “index score*”))—Embase.

(xylitol*) AND (gingivitis* OR periodontitis* OR bleeding NEXT on NEXT probing* OR gingival NEXT bleeding* OR pocket NEXT depth* OR gingival NEXT index* OR periodontal NEXT index* OR (gingival* AND index NEXT score*))—Cochrane.

The term “xylitol” was replaced with “erythritol,” “sorbitol,” “maltitol,” “isomalt,” “lactitol,” or “sugar-free” in the search for studies with other polyols besides xylitol. The searches were not restricted to chewing gum studies only.

### Study inclusion and exclusion criteria

Prospective randomized controlled clinical trials (RCTs) were included in the review. Based on our earlier systematic reviews [14, 22], we were aware of the heterogeneity of the existing studies with regard to study design, age of subjects, length of interventions, and daily polyol doses and consumption frequencies. For this reason, we decided that our evaluation would be descriptive and that a meta-analysis would not be relevant. The inclusion criterion for the aim of studies was to study the effects of sugar-free polyol gum chewing on clinical indices of gingival inflammation. An index of gingival inflammation was either the primary or secondary outcome measure in the evaluated studies. The included studies had to compare baseline or no treatment values with values obtained in the same subjects after the intervention period. The comparison/control was a sugar-free polyol gum, chewing gum base, or no product. In order to meet the inclusion criteria, the daily dose of the polyol had to be available.

Exclusion criteria used when evaluating titles and abstracts: in vitro studies; case reports; animal studies; studies in subjects undergoing orthodontic treatment; studies in patients; studies in mentally retarded or disabled subjects; studies not related to oral health; reviews, abstracts, comments, or study protocols; the polyol vehicles were candies/tablets/dragées, oral rinses or sprays, toothpastes, toothbrushes, pacifiers, milk or wipes; an index of gingival inflammation was not an outcome of the study; no control group; the study was not available in English.

**Fig. 1:**
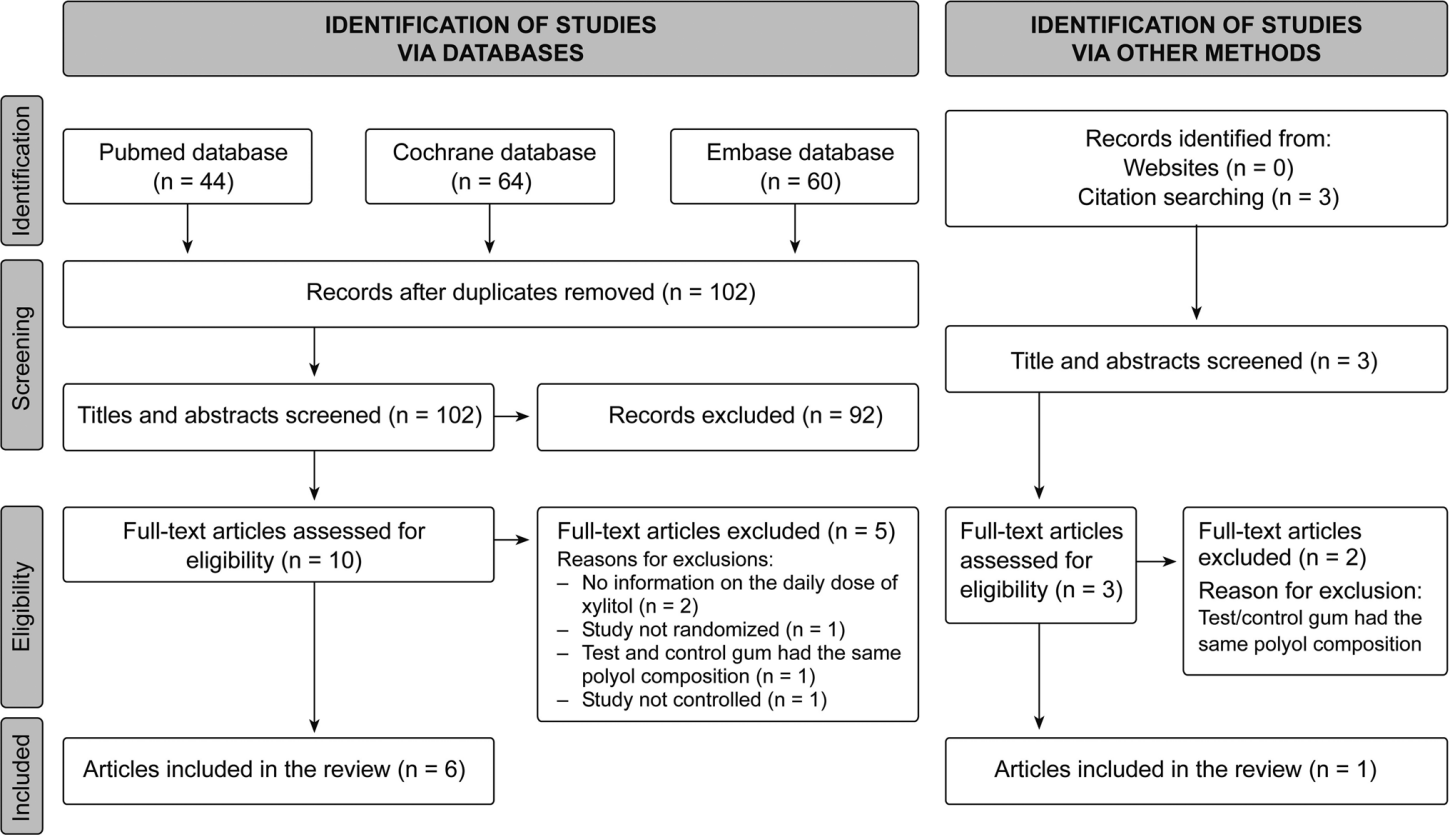
Flow chart

Main reasons for exclusion of xylitol studies when evaluating full-text articles. In three studies, the test and control gum had the same polyol composition [25–27]. In addition, one of the studies [27] did not describe randomization and blinding. Two studies provided no information on the daily dose of the polyols, study randomization, controls or blinding [28, 29]. One study was not randomized [30], and one study was not controlled [31] (Fig. 1).

### Study selection and data extraction

Screening of the records was performed after duplicate removal independently by three reviewers (ES, KP, UG). Two of them (ES, KP) have been calibrated during the evaluation and analysis process of two similar systematic reviews [14, 22]. The third reviewer (UG) is also an experienced researcher. The review team members scanned the titles and, when needed, read the abstract. The team members independently decided which studies in their opinion fulfilled the criteria for full-text review. These opinions were discussed before deciding which studies will be included in the review.

Two of the reviewers (ES, KP) collected the data from the articles chosen for the full-text review. The following data were collected: author and year of publication, study site, number and age of participants, study design, intervention and controls, assessment method, recommended gum chewing time, and main results. When data were available, one of the reviewers (ES) calculated in percentages changes in the indices within the groups, and differences in the scores between the groups at the end of the study. Any disagreements were resolved by discussion among all three authors of the systematic review. The corresponding authors were contacted via e-mail if data were missing.

### Assessment of methodological quality and risk of bias

The risk of bias of the selected articles was assessed using the Cochrane Collaboration tool for assessing risk of bias in randomized trials [32]. The three reviewers independently evaluated the included full-length articles and, based on mutual agreement, eliminated discrepancies between each individual assessment.

The studies were appraised according to the following aspects: random sequence generation, allocation concealment, blinding, completeness of outcome data, selective reporting, and funding bias. Each aspect was classified as having either low, high, or unclear risk of bias. The bias was estimated to be unclear, for example, if the study was randomized but details on randomization were not given. Also when information not found in the paper was submitted by the authors, the bias was classified as unclear. The overall level of risk for each study was classified as low (all quality items were met: high quality), unclear (unclear risk of bias for one or more domain: fair quality), or high (high risk of bias for one or more domain: low quality) [22, 32, 33].

## Results

### Study selection

In the searches, the highest number of studies was found for xylitol: when screening databases, a total of 168 titles were screened for relevance. Removing the duplicates left 102 titles to be evaluated. When full-text articles were assessed for eligibility, six articles remained to be evaluated and reviewed. In addition, in citation searching, one more article to be evaluated was found. Thus, altogether seven articles were reviewed. The flow-chart of xylitol studies is described in Fig. 1.

In the search for erythritol articles, a total of 84 titles were screened for relevance (28 PubMed, 22 Embase, 34 Cochrane). Removing the duplicates left 46 titles to be evaluated. Based on the information of the title/abstract, all articles were removed.

In the searches for sorbitol articles, 60 titles were screened for relevance (22 PubMed, 21 Embase, 17 Cochrane). The removal of duplicates left 23 titles to be evaluated. Based on the information of the title/abstract, five articles were left to be evaluated. When full-text articles were assessed for eligibility, one article remained to be reviewed. This article was also one of the seven xylitol articles to be reviewed.

In the searches for maltitol articles, 13 titles were screened for relevance (4 PubMed, 2 Embase, 7 Cochrane). The removal of duplicates left nine titles to be evaluated. Based on the information of the title/abstract, two articles were left to be evaluated. When the full-text articles were assessed for eligibility, one article remained to be reviewed. This article was also one of the seven xylitol articles to be reviewed.

In the searches for isomalt and lactitol, no articles were found. With the term “sugar-free,” new articles not found in the above searches were not detected.

### Study characteristics

All studies included in the review were prospective, randomized, controlled studies published between 1971 and 2021 [34–40]. In the seven studies evaluated, the authors reported no acute or systemic diseases for the subjects. In four studies, the participants were stated to be healthy [36, 38–40]. Two studies did not report any acute or systemic diseases, and the authors reported that the subjects had not used antibiotics prior to the study [35, 37]. In one study [34], the authors reported that the subjects were not currently receiving any medical treatment. Smoking was reported only in one of the studies; the Keukenmeester et al. [38] study had not smoking as one of the inclusion criteria. All studies reported the age of the participants (age range 18–60 years or older), sample size (ranging from 28 to 303), and study duration (from 3 weeks to 12 months). In all studies, the polyol vehicle was a chewing gum (Table 1). In three studies, the main aim was to study chewing gums with antimicrobial agents and the polyol gum was used as a control with either no gum [35] or a polyol gum as the additional control [36, 40]. The recommended daily frequency of chewing varied from two to five times, the duration from 5 to 15 min, and the daily exposure time from 15 to 50 min (Table 1).

**Table 1:** Summary of the included studies

	Subjects; *n*	Study design; outcome measure	Intervention	Comparative	Assessment method	Results
Steinberg et al. (1992); New York, USA	Adults, *n* = 28, GI < 1.5	Double-blind, randomized, controlled cross-over study (6 wk); gingival index (som)	XYL gum (1.8 g/stick*, 9 g/d*, 5xd)	SOR gum (1.8 g/stick, 9 g/d, 5xd), no gum	Gingival index CT 5 × 10 min	No change in the XYL group. GI decreased in the SOR group compared to baseline and no gum (*p* < 0.05)
Simons et al. (2001); West Hertfordshire, UK	≥ 60-yr-old adults, *n* = 164	Double-blind, randomized, controlled study (12 mo); gingival index (som)	XYL gum (289 mg XYL, 142 mg SOR, 82 mg lycasin/stick; 1.2 g XYL/d, 2.1 g polyol/d, 2xd)	No gum	Gingival index CT 2 × 15 min	At 12 mo, XYL group showed lower GI compared to no gum group (*p* < 0.001). Compared to baseline, no changes within the groups
Campus et al. (2011); Sassari, Italy	18–30-yr-old adults, *n* = 120, BOP > 25%, MS ≥ log 5	Double-blind, randomized, controlled study (30 d); bleeding score (som)	XYL gum (30% XYL, 26% SOR, 11% MAN, 1% MAL; 2.2 g XYL/d, 4.8 g polyol/d, 3xd)	Polyol mixture gum (31% SOR, 28% isomalt, 9% MAN, 1% MAL, 4.8 g polyol/d, 3xd)	Bleeding on probing CT 3 × 5 min	Compared to baseline, BOP decreased in XYL group (*p* = 0.04), no change in the control. Between-group comparison not tested
Al-Haboubi et al. (2012); London, UK	≥ 60-yr-old adults, *n* = 186	Double-blind, randomized, controlled study (6 mo); gingival index (som)	XYL gum (66%, 2.8 g/d, 2xd)	No gum	Gingival index CT 2 × 15 min	At 6 mo, XYL group showed lower GI than no gum group (*p* < 0.001). Compared to baseline, GI decreased significantly in both groups (*p* < 0.01)
Keukenmeester et al., (2014); Amsterdam, the Netherlands	> 18-yr-old adults, *n* = 303, (moderate gingivitis)	Double-blind, randomized, controlled study (3 wk); bleeding on marginal probing (pom)	XYL gum (64%*, 9 g/d*, 5xd)	MAL gum (64%*, 9 g/d*, 5xd), gum base, no gum	BOMP CT 5 × 10 min	XYL, MAL gums showed lower BOMP scores compared to gum base (*p* < 0.005) in nonbrushed lower jaw. Compared to baseline, no changes within the groups. No changes in the brushed upper jaw
Akgül et al. (2020); Istanbul, Turkey	18–29-yr-old* adults, *n* = 154	Blinded, randomized, controlled study (3 wk); gingival index (som)	XYL gum (5.4 g/d, 3xd)	Gum base*	Gingival index CT 3 × 10 min	Compared to baseline GI decreased in XYL group (*p* < 0.01), no change in the control group. At 3 wk, GI lower in the XYL group compared to the control group (*p* < 0.01)
Cagetti et al. (2020); Sassary, Italy	30–45-yr-old adults, n = 271, MS ≥ log 5	Double-blind, randomized, controlled study (12 mo); bleeding on probing (som)	XYL gum (30% XYL, 26% SOR, 11% MAN, 1% MAL; 2.2 g XYL/d, 4.8 g polyol/d, 3xd)	Polyol mixture gum (31% SOR, 28% isomalt, 9% MAN, 1% MAL, 4.8 g polyol/d, 3xd)	BOP CT 3 × 5 min	Compared to baseline, BOP decreased in XYL group (p = 0.01), no change in the control. Between-group comparison not tested

*XYL* xylitol, *SOR* sorbitol, *MAL* maltitol, *ERY* erythritol, *MAN* mannitol, *PI* plaque index, *GI* gingival index, *BOP* bleeding on probing, *BOMP* bleeding on marginal probing, *wk* weeks, *yr* years, *mo* months, *d* days, *min* minutes, *pom* primary outcome measure, *som* secondary outcome measure, *NOH* no oral hygiene, *CT* recommended gum chewing time, *details on the study obtained from the authors

In the majority of the studies, the index of gingival inflammation was the secondary outcome measure (Table 1). Only in one study the primary outcome measure was bleeding on marginal probing [38]. In two studies, the primary outcome measure was the amount of plaque [34, 35], in one plaque acidogenicity [36], in one stimulated saliva flow rate [37], in one pro-inflammatory cytokines [39], and in one caries [40]. For gingival outcome, four studies used the gingival index (GI) [34, 35, 37, 39], two studies used bleeding on probing (BOP) [36, 40], and one bleeding on marginal probing (BOMP) [38].

### Quality assessment of the selected studies

**Fig. 2:**
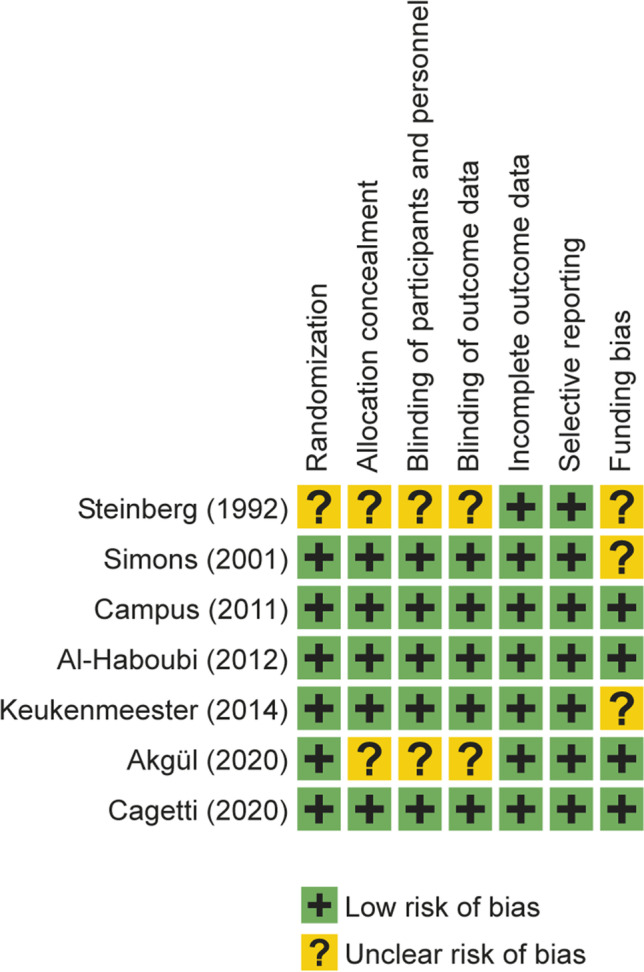
Risk of bias summary

Figure 2 summarizes the risks of bias in the evaluated studies. The risk-of-bias assessment revealed that three studies had a low risk of bias [36, 37, 40], and the rest had an unclear risk of bias [34, 35, 38, 39].

Even though we searched the literature from the early 1970s, the oldest study included in the review was from 1992 [34], and the rest of the evaluated studies had been published after 2000. This is reflected among others in the way the studies were randomized and blinded. In five studies, the participants were randomized on an individual basis using computer-generated randomization [36–40], and in one study the homes of the elderly subjects were randomized [35].

Gingival inflammation was the primary outcome of the present review, and thus, it was important that the estimation of the index reflecting gingival inflammation was performed blinded. Five of the studies gave detailed descriptions on blinding and allocation concealment; two studies merely included the statement that they were performed blinded, without providing much detail [34, 39].

The availability of individual baseline values increased the probability of finding true intervention-related changes in the indices reflecting gingival inflammation in the evaluated studies. Also, differences in changes in the gingival indices between intervention and comparison groups could be detected. In most studies, the baseline gingival indices were comparable with the post-intervention values; however, in one study, this point remained unclear [34]. The crossover design, the controls, and the long 6-week wash-out periods between the interventions should nevertheless compensate for the possible bias in the incomplete outcome data, thus presenting an unclear risk of bias [34].

All seven studies reported the results in a transparent way, making the data available for the reader. We also did not find problems with selective reporting. Three of the studies used xylitol chewing gums as controls of antimicrobial chewing gums containing chlorhexidine or magnolia extract [35, 36, 40]. These studies, however, also reported the results on the xylitol control gums and their controls even though the focus had been on the antimicrobial agents.

In three of the seven studies, the tested polyol and control chewing gums had been obtained as gifts from various companies without other apparent funding [36, 37, 39, 40]. Three studies appeared to be at least partly industry-funded, resulting in an unclear risk of bias [34, 35, 38].

### Influence of sugar-free polyol gums on gingival indices

All seven evaluated studies had a xylitol gum as one of the studied chewing gums. In five studies, xylitol chewing gum decreased significantly gingival inflammation, the effects varying from small to clinically significant. Sorbitol and maltitol chewing gums were each studied in one article [34, 38]. Sorbitol gum was associated with a small decrease in gingival inflammation [34], while maltitol gum did not decrease gingival scores when used as an adjunct to toothbrushing [38].

In three studies with fair quality, polyol gums were studied with daily doses exceeding 5 g, the recommended chewing frequencies varying from three to five times a day [34, 38, 39]. In the study by Steinberg et al. [34], a small but not significant 5% decrease in the mean gingival index was found in the xylitol gum group, while in the sorbitol gum group the 9% decrease was significant (*p* < 0.05) when compared to baseline. In the sorbitol group, the decrease was also significant compared to the no-gum control. In the Keukenmeester et al. [38] study, xylitol and maltitol chewing gums did not decrease BOMP scores compared to baseline either in the brushed or nonbrushed jaw. In the non-brushed jaw, both xylitol and maltitol gum chewing had a small but significant inhibiting effect on BOMP scores compared to gum base (*p* < 0.05) but not compared to no gum. In the 3-week study by Akgül et al. [39], xylitol gum chewing decreased the gingival index by 70% compared to the baseline (*p* < 0.01). No change was found in the gum base control group. At the end of the study, the mean gingival index was 77% lower in the xylitol group compared to the control group (*p* < 0.01) (Table 1).

In the high-quality Al-Haboubi et al. [37] study, the low mean gingival indices of elderly people further decreased by 22% in association with xylitol gum chewing for 6 months (*p* < 0.001). However, the gingival indices decreased also in the control group by 11% (*p* = 0.008). At the end of the study the mean gingival index was 21% lower in the xylitol group compared to the no-gum control group (Table 1).

In three additional studies, xylitol gum was a control of a supplemented chewing gum containing either chlorhexidine or a magnolia bark extract [35, 36, 40]. These studies had an additional control, either no gum [35] or a polyol mixture gum [36, 40], and were thus included in the present review. In the study by Simons et al. [35], xylitol gum chewing had no effect on gingival indices when compared to baseline. However, the mean gingival index was 45% lower in the xylitol gum group compared to the no-gum control group (*p* < 0.001). The magnolia bark extract studies were performed in subjects with a high caries risk [36, 40]. In the high-quality Campus et al. [36] 30-day study, the xylitol-polyol gum decreased the BOP scores by 24% (*p* < 0.04). The change in the control group was not statistically significant. The same chewing gums and chewing recommendations were also used in a high-quality 12-month study by Cagetti et al. [40]. The BOP scores decreased by 25% in the xylitol-polyol group (*p* = 0.01), while no change was seen in the control group. No between-group comparisons were available in these two studies (Table 1).

In all seven studies, except for the Keukenmeester et al. [38] study, chewing gum was used as an adjunct to normal oral hygiene. In two studies, the subjects were asked to refrain from toothbrushing in the morning of the plaque collection day [34, 38]; in five studies, no instructions were given.

### Adverse effects

Possible adverse effects connected with the studies were recorded and reported in five of the seven studies [35–38, 40]. In the magnolia bark extract study, one subject in the xylitol gum group discontinued the study based on taste disturbances [36]. In the study by Simons et al. [35], increased problems with chewing food were reported in the no-gum control group but no one discontinued the study for this reason. No other adverse effects were reported in the five studies.

## Discussion

Based on seven studies with high or fair quality, the main finding of this review is that habitual xylitol chewing gum consumption may reduce gingival inflammation. Four of the seven evaluated studies reported a within-group reduction of 21–70% in gingival scores [36, 37, 39, 40], and in one study the gingival scores were 45% lower in the xylitol group compared to the no-gum control at the end of the study [35]. The result was similar in long-term studies lasting several months [35, 37, 40] and in short-term studies lasting a few weeks [36, 39]. Only one sorbitol and maltitol article fulfilled the inclusion criteria [34, 38]; no clinical erythritol chewing gum studies were found in the literature searches.

The majority of xylitol chewing gum studies agree that chewing gums with a high xylitol content and daily doses of 5 g or more are needed for “xylitol-effects,” demonstrated, for example, in association with decreases in counts of *mutans streptococci* [14, 41]. Surprisingly, only one of the three studies conducted with such daily xylitol doses showed not only statistically but also clinically significant decreases in the gingival scores [39], while two studies showed no decreases [34, 38]. The four studies conducted with chewing gums with rather low daily doses of xylitol showed significant reduction of gingival inflammation compared to baseline values [36, 37, 40] or lower scores in the xylitol group compared to the control in the end of the study [35]. Even though most xylitol studies support the idea of a dose–response for the beneficial effects of xylitol, some studies have reported decreases in counts of *mutans streptococci*, plaque accumulation and gingival indices with rather low daily doses of xylitol [42, 43]. Interestingly, in the two Italian studies in which a xylitol-polyol gum with 44% xylitol was compared with a sorbitol-polyol gum, the xylitol-polyol gum significantly decreased BOP scores by 24–25%, while no significant changes were found in the sorbitol-polyol control groups [36, 40]. The result is in line with earlier chewing gum studies demonstrating that xylitol gum chewing reduces plaque formation and *mutans streptococci* counts compared to chewing gums with sorbitol as the sweetener [14, 22]. It can only be postulated how high-concentration xylitol chewing gums with higher daily xylitol doses might have performed in these two studies.

There may be so far little evidence supporting the use of sugar-free polyol chewing gums in reducing gingival inflammation, but the results on supplemented xylitol chewing gums certainly should inspire researchers to do more high-quality studies on the topic [24]. For example, a maltitol-xylitol chewing gum supplemented with green tea significantly reduced gingival inflammation [27]. In the study by Simons et al. [35], the chlorhexidine gum was superior to the xylitol gum in reducing gingival scores, even though the xylitol gum performed better than the no-gum control. In the study, no adverse effects related to the supplemented gum were reported; however, chlorhexidine is a broad-spectrum antimicrobial agent which has been associated with, for example, taste disturbances and allergies [44]. Two of the studies we evaluated aimed at studying supplemented chewing gums, and polyol gums were used as controls [36, 40]. In these Italian studies [36, 40], the magnolia bark extract gum was superior to the two control gums, the xylitol-polyol and the sorbitol-polyol gums. When the controls were compared, the xylitol-polyol gums reduced gingival scores better than the sorbitol-polyol controls. Magnolia bark extract has antimicrobial activity against oral biofilms [45]. The extract is used in Chinese medicine, and it has the status of a novel food in Europe [40]. The systematic review by Keukenmeester et al. [23] found positive effects on gingival inflammation for both magnolia and chlorhexidine chewing gums. The recent review by Muniz et al. [24], however, reached the conclusion that there is no robust evidence for the clinical indications of sugar-free chewing gum as adjunct to toothbrushing for the treatment of gingivitis. An interesting aspect of future studies could be synergy between xylitol and the antimicrobial agent in the chewing gum. Unfortunately, the study designs of the above three studies included in this review do not allow any conclusions on synergy to be drawn.

Recently we reviewed effects of habitual xylitol gum chewing on plaque formation and concluded that habitual xylitol gum chewing is likely to decrease plaque [22]. In that review, we found 424 studies for screening of titles and abstracts, while in the present review, the number was only 102. This suggests that plaque formation has been studied mainly from a cariological viewpoint. Dental plaque is a risk factor for gingival inflammation; thus, the results of the earlier and present review were expected to be similar. Four of the evaluated studies were included in the earlier review [34, 37–39]. In two of them, a significant decrease was seen both in the plaque and gingival scores [37, 39]. In the Steinberg et al. [34] study, plaque decreased both in the xylitol and sorbitol groups, while the gingival scores decreased only in the sorbitol group. In the Keukenmeester et al. [38] study, the plaque indices decreased both in the xylitol and maltitol groups in the brushed upper jaw, while no changes were observed in the gingival scores. In the Simons et al. [35] study, the plaque scores decreased in the xylitol group but not the control group, while for gingival scores no within-group changes were observed. However, both the gingival scores and plaque indices were lower in the xylitol group compared to the control group at the end of the study. In the two Italian studies [36, 40], plaque formation was not assessed, but the decrease of *mutans streptococci* counts in the xylitol-polyol groups suggests that the plaque may be less adhesive/virulent [46]. Clearly, changes in plaque scores in the above studies were not always reflected in the gingival scores. In our earlier review [22], we concluded that xylitol gum showed specific reducing effects on plaque accumulation. In the present review, the inconsistent results of the studies does not allow such conclusions with regard to gingival inflammation.

Although plaque accumulation in the above studies was not always reflected in gingival scores, xylitol should influence gingival scores with the same mechanisms that influence plaque accumulation. Xylitol consumption has reduced the acid production potential of plaque [47], thus not favoring acidogenic and aciduric microorganisms like *mutans streptococci*. There is good evidence to suggest that habitual xylitol consumption reduces *mutans streptococci* counts in plaque [14], which could result in less adhesive plaque. It has also been suggested that a xylitol-induced decrease in the extracellular polysaccharides could reduce plaque [22]. Interestingly, xylitol has inhibited biofilm formation of wound-infection pathogens like *Pseudomonas aeruginosa*, even though its growth is not inhibited by xylitol. In these studies, it was suggested that xylitol would inhibit the extracellular matrix formation [48]. This mechanism of action has been proposed for xylitol, but there is a lack of studies with regard to the effects of other polyols.

In our earlier review, we presented the idea that chewing time may be an important confounding factor in xylitol chewing gum studies [22]. Xylitol is easily soluble and shows no retention to plaque; thus, it may be important for the mechanism of action of xylitol that oral clearance is not too rapid. Xylitol dissolves from a chewing gum with a high concentration peak in the saliva, the bulk of the xylitol (and sweetness) being dissolved at 3 min of gum chewing [49]. Thus, short chewing times resulting in high xylitol levels in the plaque may be of importance. This idea has been presented only for xylitol [22] but may apply also to other polyols. The rather long 10-min chewing time could explain why the polyol chewing gums had such minor effect on gingival scores in the Steinberg et al. [34] and Keukenmeester et al. [38] high-polyol-dose studies. On the other hand, in the Akgül et al. [39] study, chewing xylitol gum for 3 × 10 min with a daily xylitol dose of 5.4 g showed large decreases both in gingival scores and plaque accumulation in the xylitol group. The two Italian studies were the only studies included in the review with short recommended chewing times, 3 × 5 min per day [36, 40]. It could be postulated that the short chewing time may have boosted the effects of the xylitol–polyol mixture gum in the studies at low daily doses of xylitol. Also, the recommendation to refrain from toothbrushing for 1 h after gum use may have affected the results. Clearly, since chewing time may influence the outcome of chewing gum studies, it could be an interesting research aspect of future chewing gum studies.

To our surprise, there were only a few studies on the effects of xylitol gum but also of other sugar-free polyol gums on gingival health. To our knowledge, no studies have been published on erythritol chewing gums. In the only sorbitol gum study evaluated in this review, sorbitol gum chewing showed a small reduction in gingival and plaque scores [34]. Most studies with sorbitol gum have demonstrated no decreases or small but significant decreases in plaque accumulation, the effects being smaller than those found for xylitol [22]. In the evaluated maltitol study, a high-concentration maltitol chewing gum was used as an adjunct to toothbrushing. Gingival inflammation did not decrease, but a small decrease was found in plaque scores [38]. The few existing clinical studies suggest a decrease in plaque scores in association with consumption of high-concentration maltitol products [50, 51]. Sorbitol and maltitol are considered microbiologically rather inert but they are sweet and, thus, add to the saliva secretion-enhancing effect of gum base. Studies using gum base as the control demonstrate that the plaque-reducing effects of polyol gums may not be attributed to chewing per se [22].

Adverse effects were registered in five out of the seven reviewed studies and reported only in two studies [35, 36]. In the Campus et al. [36] study, one subject in the xylitol gum group discontinued the study based on taste disturbances, and in the Simons et al. [35] study, the control group experienced increased problems with chewing food. The studies reported no problems with temporomandibular joint dysfunction even though it might have been expected since in two of the studies the subjects were older people [35, 37]. In contrast, in the Simons et al. [35] study, the elderly subjects in the xylitol gum group reported significant improvement in ability to taste and chew without problems. Also the elderly subjects of the Al-Haboubi et al. [37] study reported no TMD problems but a significant improvement in self-perceived oral health in the xylitol gum group. The polyol sweetener could also have been associated with adverse effects. All polyols of the evaluated studies belong to FODMAP (fermentable oligo-, di-, monosaccharides and polyols) substances which may not suit persons with a tendency for digestive disorders. No such adverse effects connected with the polyol sweeteners were reported even though in one study the daily dose of xylitol/maltitol was rather high [38]. In fact, complaints about digestive discomfort in xylitol studies are rare [14, 52].

The present review identified seven xylitol chewing gum studies, one sorbitol chewing gum, and one maltitol chewing gum study with either high or fair quality. Based on the results of five studies, it appears that habitual use of xylitol chewing gum as an adjunct to toothbrushing may decrease gingival inflammation in adults both in short-term and long-term consumption; however, two studies found no effect. As for sorbitol and maltitol, only sorbitol gum chewing showed a small decrease in gingival scores. The effects of sugar-free polyol chewing gums on gingival inflammation clearly need further, well-controlled studies.
